# Comparison of Check-All-That-Apply (CATA), Rate-All-That-Apply (RATA), Flash Profile, Free Listing, and Conventional Descriptive Analysis for the Sensory Profiling of Sweet Pumpkin Porridge

**DOI:** 10.3390/foods12193556

**Published:** 2023-09-25

**Authors:** DaEun Kim, HanSub Kwak, Manyoel Lim, Youngseung Lee

**Affiliations:** 1 Department of Food Science and Nutrition, Dankook University, Cheonan 31116, Republic of Korea; toffl0716@naver.com; 2 Food Processing Research Group, Korea Food Research Institute, Wanju-gun 55465, Republic of Korea; hskwak@kfri.re.kr (H.K.); manyoell@kfri.re.kr (M.L.); 3 KFRI School, University of Science and Technology, Wanju-gun 55465, Republic of Korea

**Keywords:** rapid sensory profiling, consumer-based methods, conventional descriptive analysis, Smith salience index

## Abstract

With significant progress in the use of rapid descriptive methodologies as alternatives to conventional descriptive analysis (DA), several consumer-based approaches have emerged. In this study, we compared four such methodologies—check-all-that-apply (CATA), rate-all-that-apply (RATA), flash profile (FP), and free listing (FL)—for sensory profiling to DA, using six sweet pumpkin porridges. The DA involved eight trained panelists, whereas each consumer evaluation engaged 60 untrained consumers. Overall, the performance of the consumer methods was similar to the DA, and it could effectively profile differences in consumer perceptions of sensory attributes, as evident from high regressor vector (RV) values (>0.89). RATA exhibited the highest similarity to the DA (Rv = 0.96), featuring quicker and less tedious processes compared with FP or FL. Novel combined methods for sensory characterization using the strengths of these four approaches are warranted. This includes leveraging the simplicity and versatility of CATA or RATA coupled with the capacity of FP or FL to capture spontaneous perceptions of products by consumers.

## 1. Introduction

The sensory profiling technique is an essential tool for the comprehensive description of the sensory characteristics of a product. It is widely used in the food industry because of the several advantages it offers; for example, it helps to understand consumer acceptability criteria, ensure product quality, guide product innovation, and guarantee customer satisfaction [1,2]. Such profiling provides guidance on the sensory properties that a product should possess. It also plays a vital role in the development and marketing of new products, repositioning of existing products, optimization of manufacturing processes, and quality management in the food industry [3,4,5].

Conventional descriptive analysis (DA) is widely recognized as the most sophisticated method for profiling the sensory characteristics of a product [6]. It is a systematic and objective strategy in which trained sensory panelists assess products based on its sensory attributes, ensuring the accuracy and reproducibility of data [7,8,9]. However, DA has some drawbacks in that it requires significant time and effort to generate reliable results. Recruiting or retraining panels for product testing can pose challenges, which particularly depend on a company’s size and the specific circumstances faced by it [9,10].

Pineau et al. [11] reported that approximately nine hours, on average, need to be spent on DA, starting from the definition of sensory terms to the training of panels for evaluating a sample set of nine products that exhibit moderate differences. Moreover, trained panels may overlook attributes that are crucial to consumers or may perceive attributes that consumers cannot detect, resulting in inconsistencies between consumer perception and evaluation by trained panels [12].

In view of the significance of reflecting consumer perception in product development and considering the limitations of DA stated above, rapid sensory profiling methods based on consumer perception have been developed. These methods aim to expedite new product development and provide a more accurate reflection of consumer perception regarding the sensory properties of food. Representative rapid sensory profiling methods include projective mapping (PM) [13], polarized sensory positioning (PSP) [14], pivot profile [15], free-choice profiling (FCP) [16,17], sorting [11], flash profiling (FP) [18,19], Napping^®^ [20], check-all-that-apply (CATA) [21], rate-all-that-apply (RATA) [22], and free listing (FL) [23].

Comparative studies on product configurations, descriptions, and discrimination between DA and consumer-based methodologies have been actively conducted. Some examples of such comparisons include those between DA and CATA for orange-flavored powdered beverage samples [24]; between PM and CATA for brewed black coffees [25]; between pivot profile and CATA for instant black coffee [26]; among RATA, CATA, and sorting and Napping^®^ for plant-based milk [11]; between CATA and FCP for fish [17]; among DA, FCP, and PSP for water [14]; and among CATA, FP, and FL for RTD coffee beverages [27]. Among various consumer-based methods, studies on FL as an alternative to DA are limited, although this method has gained increasing attention owing to its ability to reflect the spontaneous perception of the tested products held consumers [23]. FL is predominantly used in anthropological research to conduct cultural domain analysis but has increasingly been applied in consumer science research [28]. This method allows consumers to freely describe the characteristics of a product after tasting it.

Yoon et al. [27] compared the performance of three rapid sensory profiling methods, namely, CATA, FP, and FL, in evaluating RTD coffee beverages and investigated their potential as alternatives to DA. In their analyses, the three consumer-based methodologies demonstrated potential as viable substitutes for DA when assessing variations in consumer perceptions regarding sensory attributes of RTD coffee beverages exhibiting moderate to large sensory distinctions. They also highlighted that RATA could be considered as an alternative, noting its superior effectiveness over CATA in evaluating highly similar products. This superiority is primarily attributed to the advantages offered by RATA in quantifying attribute intensities [29].

Porridge is a semisolid food prepared by pouring water over grains, such as cooked rice, barley, and beans, followed by extensive boiling until the starch in the grain is fully gelatinized [30]. With the increased consumption of convenience foods over time, products processed into home meal replacements (HMRs) have emerged, which are a form of porridge that has been transformed into a convenient meal option that does not require prolonged cooking.

The instant porridge market in South Korea has consistently grown by over 25% since 2016, and it reached KRW 88.5 billion in 2018 [31]. According to a consumer survey in 2019, the most mentioned porridge varieties were pumpkin porridge (38.8%), abalone porridge (31.4%), and vegetable porridge (25.5%), indicating that consumers have the highest preference for pumpkin porridge [32]. Pumpkin belongs to *Cucurbitaceae* and is rich in dietary fiber, carotenoid compounds, β-carotene, proteins, vitamin A, vitamin E, and other nutrients [33,34]. The intake of these nutrients in significant amounts enhances immune function and reduces the risk of heart disease and cancer [34]. These health benefits have prompted increasing interest in pumpkin as a functional ingredient, and these advantages are recognized not only in South Korea but also globally [33,34,35]. With the continued growth of the instant porridge market and the added functional benefits of pumpkin, the market potential for instant pumpkin porridge needs to be explored.

Despite its multiple functions, the product attributes that significantly influence consumer perception of sweet pumpkin porridge have scarcely been investigated. Therefore, in this study, we aimed to compare the effectiveness of four rapid sensory profiling methods (CATA, RATA, FP, and FL) in evaluating sweet pumpkin porridge. We also explored the potential of these methods as alternatives to the conventional DA.

## 2. Materials and Methods

### 2.1. Samples and Preparations for Sensory Evaluation

Six brands of commercially available sweet pumpkin porridge HMR products were used in this study. A description of the products, which were purchased at local supermarkets, is presented in Table 1.

All products were stored at 20~22 °C until evaluation, following the manufacturer’s recommendations. During the evaluation, each sample was heated for the recommended heating time. They were stored in a thermos bottle (DAISO CS Stainless Handle Water Bottle (1.6L)-1003054, Chef Star Co., Ltd., Gimpo-si, Republic of Korea) and maintained at 30 ± 5 °C until served. The samples were provided in halves of 70 mL plastic multipurpose cups coded with a three-digit random number and served on a white plastic tray with water, a spit cup for expectoration, and a plastic spoon. The water provided was used to clean the mouth between the partaking of samples. This study was approved by the Institutional Review Board of Dankook University in Korea (IRB No. DKU 2022-06-031).

### 2.2. Descriptive Analysis (DA)

#### 2.2.1. Panel Training

On the first day of training, the panelists were instructed to gain an understanding the goal of the DA, the usage of scales, and the quantification of the intensity of sensory attributes. From the second to fifth days, the panelists were trained to detect and quantify the intensity of appearances, aroma, flavors, and texture attributes for various food products. Aroma was defined as the scent, or smell, of samples before consumption, whereas flavor was the combined sensory perception of the taste and aroma during consumption. During the 6th day of training, the panelists were instructed to identify and define the sensory attributes, such as aroma, taste, and texture, of the sweet pumpkin porridge samples using terms perceivable and understandable to each of the panelists. On the 7th day of training, the panelists developed a final glossary of descriptions, consisting of 20 attributes, and established a final lexicon (Table 2). On the 8th day of training, the panelists were trained to become familiar with the sensory terms they created, and they developed references for each attribute and determined the appropriate intensities for each reference. Each training session lasted approximately one hour per day.

#### 2.2.2. Descriptive Analysis Evaluation

The sensory panel consisted of eight trained assessors (7 females and 1 male, aged 20–30 years) recruited from Dankook University (Cheonan-si, Republic of Korea). The panelists were selected based on their interest in the evaluation, ability to perceive basic aroma and taste, potential to express characteristic terms objectively and diversely, willingness to participate, and food allergy status. All panelists signed consent forms and provided informed consent prior to the evaluation. They received a small compensation after the evaluation.

The evaluation was conducted at a location that was free from unpleasant odors and ambient noise from machinery systems, such as refrigerators, air conditioners, and equipment. Additionally, the evaluation was conducted in independent individual sensory booths that allowed for the isolation of the panelists. The evaluation took place under simulated daylight provided through artificial lighting at 22–24 °C and 50–55% relative humidity, ensuring air circulation [36]. The panelists were instructed to refrain from eating food, except for brushing their teeth and drinking water, starting 2 h before the test time. The test samples were presented to the panelists in duplicate, following a randomized complete block design in which the block represented the replication. A total of 20 attributes representing appearance, aroma, flavor, and mouthfeel were evaluated based on a 16-point numerical scale (0 = “not present”, 1 = “very weak”, and 15 = “very strong”) with references (Table 2). The panelists were instructed to refresh their palate with water between samples. A 10 min break was allocated between each set of replications. Consumer data were collected using paper questionnaires.

### 2.3. Consumer-Based Sensory Evaluation

#### 2.3.1. Consumers

For the consumer tests, participants were recruited either offline from the university campus or online, based on their interest in participation. Only those who consumed sweet pumpkin porridge at least once a month, on an average, were selected for this test. Individuals with allergies to sweet pumpkin porridge and dairy products were excluded from the tests. A total of 240 untrained consumers between the ages of 20 and 60 participated in four consumer tests, with 60 consumers for each test. Consumers were randomly assigned to one of the four consumer methods. A between-subject design was used to compare the four methods. The consumer tests were conducted in the same location where the DA was previously carried out. All participants signed consent forms and provided informed consent prior to the evaluation. They received a small amount of compensation after the evaluation.

#### 2.3.2. Check-All-That-Apply

Sixty consumers (49 females, aged 20–50 years) participated in the CATA method. For the CATA questionnaire, 20 terms were selected based on the attributes generated in the DA and in previous studies [37] describing the sensory attributes of sweet pumpkin porridge. For each sample, the consumers were asked to check all terms that they considered appropriate to describe each sweet pumpkin porridge sample from a list of 20 terms. The order of the samples in the questionnaire was balanced within and across participants using a Williams design to avoid order bias. To minimize any influence of the previous sample tested, water for rinsing the mouth was provided.

#### 2.3.3. Rate-All-That-Apply

Sixty consumers (51 females, aged 20–50 years) participated in the RATA method. The RATA questionnaire used the same 20 terms as the CATA questionnaire for future comparison of the two methods. Consumers were asked to check all of the terms that they considered to be appropriate to describe the samples and then rated the intensity of the relevant terms using a 5-point intensity scale (1 = “low intensity”, 3 = “medium”, and 5 = “high intensity”). The consumers were instructed to leave the scale blank for terms that were not relevant in describing the samples. The order of the samples in the questionnaire was balanced within and across participants using a Williams design to avoid order bias. Water was provided for rinsing the mouth between the testing of each sample to minimize the influence of the previous sample.

#### 2.3.4. Flash Profile

Sixty consumers (47 females, aged 20–50 years) participated in the FP method, which comprised the following two evaluation sessions:

Session 1: The consumers were presented with the samples simultaneously, and the serving order followed a Williams Latin square design. Each consumer individually generated a list of attributes perceived before, during, and after eating the samples. The consumers were also asked to generate attributes that could differentiate the six samples from one another. They were also requested to derive at least five attributes and as many as they could for the samples. They were instructed to avoid hedonic or unrelated sensory terms for the samples. Once all the consumers had completed the session, a 10 min break was introduced before proceeding to the second session.

Session 2: The consumers were provided with all six samples simultaneously. They were asked to rank each product based on the generated terms using the individual attributes created during session 1. This was conducted by marking the position of the sample on the 15 cm line scale. The median value of the line was indicated by a point. Marking the sample more toward the left relative to the center point indicated perceiving the attributes to a lesser extent, whereas placing it more toward the right indicated a stronger perception. In cases where samples had the same intensity as the attributes, consumers were instructed to circle the tie samples to allow for tie rankings to avoid confusion. During the evaluation, consumers were free to aroma or flavor the samples as needed.

#### 2.3.5. Free Listing

The FL method was conducted with 60 consumers (43 females, aged 20–50 years). The consumers were provided with a 1/2-size space of the questionnaire per sample and were free to fill out all sensory terms related to each sample without any special restrictions; however, expressions comparing the samples to be evaluated were not allowed (i.e., 365 samples are more yellow than 214 samples). To minimize the influence of the previous sample tested, water for rinsing the mouth was provided. The assessors were given the following instructions orally:

“Write down all the attributes you perceive about each product, excluding emotions and subjective opinions. There is no time limitation, and you may consume the sample whenever you wish. Please remember that this is an individual task, so avoid interacting with your fellow assessors.”

### 2.4. Feedback Questionnaire

Consumers who completed each consumer-based method were asked to rate the difficulty (1 = extremely easy, 3 = normal, and 5 = extremely difficult) and degree of tediousness (1 = extremely not tedious, 3 = neutral, and 5 = extremely tedious) of each method performed on a 5-point scale. We also collected the time spent on the evaluation by requesting the consumers to document the start and end times of the evaluation.

### 2.5. Data Analysis

The DA data were analyzed using an ANOVA (sample as a fixed effect and panelist as a random effect) to determine the significant differences among the samples at a 5% significance level [29]. In the case of significant differences, a post-hoc test was performed using the Tukey’s honest significant difference test (HSD) at a 95% confidence level. Principal component analysis (PCA) was performed to identify the relationship between samples and attributes and to the find similarity among samples. A PCA can provide consensus construction and can be used to compare the proximity between terms used by other evaluators to describe the product [38]. Only attributes assessed as significantly different using ANOVA were used for the PCA and evaluated on average scores using a Pearson correlation matrix (*p* < 0.05).

For each term, significant differences in the CATA counts among samples were determined by applying the Cochran’s Q test (*p* < 0.05), which is a nonparametric statistical test used to analyze the significance of differences in the proportions or counts. Correspondence analysis (CA), using the significant attributes determined with the Cochran’s Q test, was performed to display the product configuration [39]. CA is a generalized PCA suggested for the analysis of qualitative data [40]. It allows for the visualization of similarities among products, similarities among descriptors, and associations between descriptors and products [41]. However, the perceptual map resulting from the CA displays only two (rarely three) dimensions, which can lead to an incomplete determination of the relationships between properties and products. Therefore, we also performed a multiple discriminant analysis (MDA) to assess the connection between the sensory descriptors and individual samples. The analysis involved computing the cosine of the angle formed between each attribute and sample, with values ranging from −1 to +1. This approach facilitated the identification of attributes that exhibited a significant association with each sample and provided a comprehensive understanding of the relationship between products and attributes. A relationship is considered minimal when the absolute cosine values, denoted using the equation 0.707 (=cos (45°) = −cos (135°)), are below this threshold [42].

For the RATA data, the mean RATA scores were computed by considering the numbers assigned in the ascending order of intensity on a scale from 1 (low) to 5 (high); any term that was not selected by consumers was treated as 0, thus, rendering this a six-point scale (0 to 6) [43]. An ANOVA followed by a post-hoc Tukey’s HSD test was conducted at a 95% confidence level. PCA was employed to analyze the sample configurations using the means of the RATA scores. Only terms that were significantly different between samples were included in the PCA (*p* < 0.05).

The FP data were pretreated before analysis; the pretreatment involved correcting grammar and excluding unusable terms (nonsensory or symbol terms). The purpose of the pretreatment was to refine and filter the attribute terms provided by the consumers [44]. After the pretreatment, ranking data for each attribute were manually collected and analyzed using the generalized procrustes analysis (GPA) [8]. GPA does not necessarily require an equal number of response variables across different datasets. This enabled us to obtain a consensus configuration of sensory maps for the assessors [3,45].

The FL data were subjected to the same pretreatment process as the FP data. The total number of descriptors mentioned by all respondents and the minimum, maximum, and average positions mentioned by consumers were calculated for each sample [46]. The sensory descriptors for each sample were analyzed using R programming. For this analysis, only the sensory attributes mentioned by at least 10% of the consumers were included. The item occurrence frequency ratio was calculated based on the average rank of each item for all participants. The resulting Smith salience index (SSI) is a statistical index used in FL analysis to quantify the salience or importance of each item, as indicated by the participants [23]. Subsequently, a PCA was conducted using the results obtained from the SSI analysis.

Multiple factor analysis (MFA) was conducted to compare the data obtained from DA and four consumer-based sensory profiling techniques. The factors derived from the MFA were used to calculate the Rv coefficient, which determined the level of similarity among the profile datasets. The Rv coefficient acts as a multivariate extension of the correlation coefficient and varies between 0 and 1. Values closer to 1 indicate a higher degree of similarity [47]. All statistical analyses were performed using the XLSTAT software version 2016 for Windows (Addinsoft Inc., Paris, France), except for the SSI analysis, which was conducted using the Anthrotool package in R version 2.12.1 (R Development Core Team, 2010).

## 3. Results and Discussion

### 3.1. Descriptive Analysis

Twenty sensory attributes were used in the DA. The ANOVA results, as well as the average intensities of each attribute for the samples, are presented in Table 3. The ANOVA revealed significant differences among the samples for all sensory attributes (*p* < 0.05). The intensity of the “yellow color” was the highest in P5 and P6, whereas these samples showed the lowest intensity of the “brown color”. Although detailed information on the manufacturing process for each sample was not disclosed, these results might be due to the different extent of browning caused by the caramelization of sugars present in the pumpkin porridge. This reaction can vary depending on the temperature and duration of heating [48]. Furthermore, high-temperature processing not only influences the browning reactions but also results in a decrease in the content of volatile aroma compounds in food products [49,50]. Therefore, it is hypothesized that P3, with a higher value of “brown color”, could potentially exhibit lower values for most of the aroma attributes (Table 3). “Sweet pumpkin aroma” and “sweet pumpkin flavor” were the highest in P2, despite its low content (26.4%) of sweet pumpkin compared with other samples (P3, P4, and P5 with 40% or greater content of sweet pumpkin). We believe that additional flavor agents or seasonings might have been added to P2 to enhance the sweet pumpkin aroma or flavor.

The “sweet flavor” was higher in P1, P2, and P6 than in the other samples. These samples had a sugar content of 30% or more, as indicated by the nutritional labels. P4 exhibited the most pronounced intensities of “sour aroma” and “sour flavor” in the samples. While specific details on the product formulations remain undisclosed, the sourness in this product could potentially be attributed to the presence of added organic acids. Notably, this product contains the highest proportion of sweet pumpkin (48.9%, Table 1) compared with all of the other samples tested. The use of organic acids to lower the pH may contribute to an extended shelf-life of this sample [51]. P4 also showed the highest “smooth swallowing” among the samples, and this could be due to the xanthan gum added to this product. Xanthan gum offers the advantages of dispersing and hydrating well at room temperature, and it plays a role in modifying the strength of the mucosal adhesion of food, thereby, enhancing the formation of boluses and lubrication by saliva. Therefore, xanthan gum is considered beneficial for dysphagia food formulations that require a smooth swallowing experience, and it is commercially used in pureed foods [52]. The highest values for “residual” and “graininess” were observed for P1. This could potentially be attributed to the presence of diverse diced ingredients, including 8% sweet pumpkin dice and 4.2% chestnut dice, incorporated as additives (Table 1).

**Figure 1:**
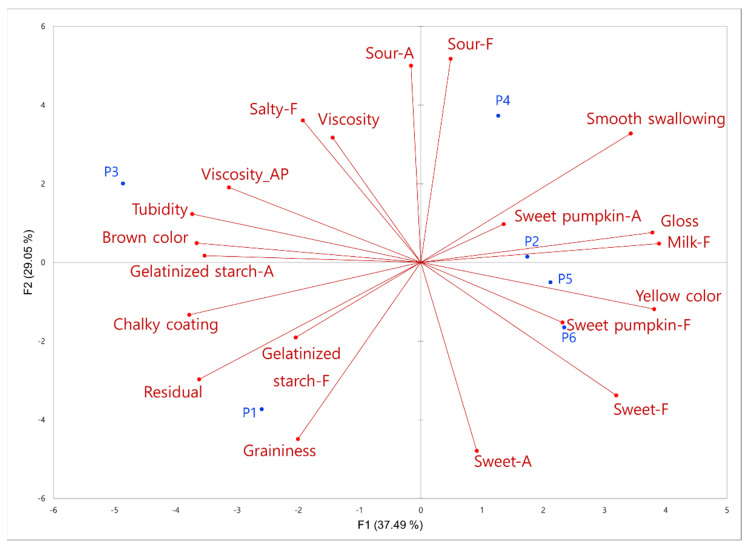
Principal component analysis of 20 descriptive sensory attributes (product codes as in Table 1). AP, F, and A at the end of attribute indicate appearance, flavor, and aroma, respectively.

The PCA was performed using the attributes that showed significant differences (*p* < 0.05) (Figure 1). The first and second principal components (F1 and F2) accounted for 37.49% and 29.05% of the variance in the experimental data, respectively. As shown in Figure 1, the samples P2, P5, and P6 were grouped together with attributes of “yellow”, “sweet pumpkin flavor”, and “sweet pumpkin aroma”, whereas the sample P3, located in the upper left of the map, could be characterized with “brown”, “starch aroma”, “turbidity”, and “appearance viscosity”. Samples P4 and P1 were clearly divided into two groups in the opposite direction based on texture attributes.

P1 was characterized by “residual”, “graininess”, and “gelatinized starch flavor”, whereas P4 was characterized by “smooth swallowing”. Interestingly, P4, the sample with the highest sweet pumpkin content, did not show any significant correlation with the “sweet pumpkin” flavor and aroma. This indicates that a higher sweet pumpkin content does not necessarily result in a stronger perception of sweet pumpkin flavor and aroma. This once again supports the notion that the sweet pumpkin porridge sample lacks potent volatile and aromatic constituents.

### 3.2. Check-All-That-Apply

Significant differences between samples in terms of the frequency of use for each sensory term are presented in Table 4 (*p* < 0.05). Of the 20 CATA attributes, most attributes, with the exception of “sweet_A” and “milk_F”, exhibited significant differences between the samples. This suggests that consumers were capable of distinguishing between sweet pumpkin porridge samples based on their perceived intensity of sensory characteristics. The robustness of CATA in providing reliable insight into the sensory characterization and the ability of the method to effectively discriminate between samples is well documented [53].

Furthermore, significant correlations were observed between the intensities of the sensory characteristics obtained by the trained panelists and the frequencies from CATA for all sensory terms, except “viscosity” (*r* = 0.62, not significant). The weak correlation observed for “viscosity” could be attributed to P4, which received the highest score in DA but had the lowest frequency in CATA. Ares et al. [54] reported that strong correlations between rapid sensory profiling and DA could be achieved for sensory attributes that are widely recognized or are easily perceivable by consumers. “Viscosity” is a difficult attribute for untrained consumers to describe and is considered one of the complex texture characteristics [55,56].

**Figure 2:**
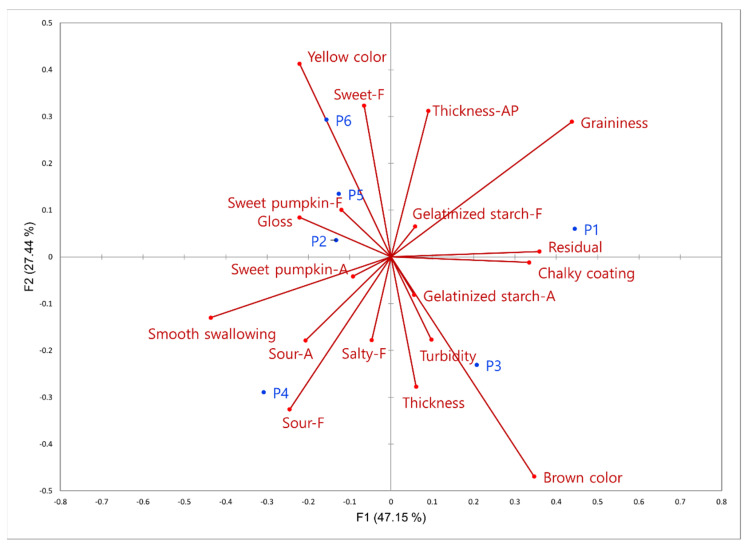
Correspondence analysis (CA) plot for the significantly different check-all-that-apply (CATA) characteristics. AP, F, and A at the end of attribute indicate appearance, flavor, and aroma, respectively.

As shown in Figure 2, CA, which is an effective tool for analyzing and visualizing relationships between sensory attributes and samples, could explain more than 70% of the variance in two dimensions. For F1, P1 showed a strong correlation with “graininess”, whereas P4 was closely related to “sour_F”, “sour_A”, and “smooth swallowing”. For F2, P3 exhibited a strong correlation with “brown color” and was positioned nearby. Additionally, P6 showed a significant association with “yellow color”, whereas P2, P5, and P6 were grouped together. These findings exhibit a strong alignment with the configurations of the samples and attributes, as well as the clusters observed in the DA PCA plot (refer to Figure 1).

An MDA was performed to assess the degree of association between products and attributes based on the angle between cosines in the perceptual map (Table 5). MDA has the capability to establish direct correlations between attributes and samples, a feature not always achievable with the visualization of CA using a two-dimensional map [3]. The cosine values range from −1 to +1, whereby absolute values below 0.707 suggest minimal spatial association [42].

Table 5 shows the relationship between the sensory attributes and the samples using the MDA. Absolute values above 0.707 indicate a strong relationship between the samples and attributes. These results are consistent with the CA results, and provide the relationships between samples and their sensory terms based on numerical values. For “turbidity”, it is possible to accurately differentiate between P3 and P6, and it is evident that P5 was not characterized by “thickness_AP”. In other words, P5 can be perceived as having a lower appearance viscosity. Among the samples, P1 exhibited the largest number of significant sensory attributes (*p* < 0.05), whereas P2 had a strong correlation with only 2 of the 20 sensory attributes. This highlights that P2 had significantly fewer sensory attributes.

In addition, P1 and P4 were differentiated by a significant number of attributes. Interestingly, P1 had the lowest pumpkin content among all of the tested samples, whereas P4 had the highest. Unexpectedly, these samples were differentiated by attributes other than pumpkin-related flavor or aroma. P1’s low pumpkin content might have led to the addition of additives to enhance its sweetness, which could explain the significant relationship of “sweet_F”.

### 3.3. Rate-All-That-Apply

The results of the ANOVA conducted on the RATA data showed that of the 20 attributes, 16, except for “sweet_A”, “gelatinized starch_A”, “salt_F”, and “gelatinized starch_F”, exhibited significant differences (*p* < 0.05) (Table 6). In comparison to the CATA results, fewer attributes showed significant differences. Similar to the CATA results, no significant differences were observed for “sweet_A”. This could imply that aroma attributes might be more challenging for consumers to discriminate compared with flavor attributes [57]. Furthermore, “gelatinized starch_A” and “gelatinized starch_F” were the main attributes in the DA, whereas they did not show significant differences in the RATA. Ares et al. [54] reported poor correlations between consumer-based sensory profiling and DA for the attributes that are not commonly definable, such as salami scent. Attributes that are widely recognized, such as basic tastes, displayed robust correlations between these two methods. In the present study, the evaluation of “gelatinized starch_A” and “gelatinized starch_F” of the samples might have been challenging for the consumers. Previous research concluded that the term discrimination between CATA and RATA was similar. We also found that the term discrimination results between the CATA and RATA methods were not significantly different (*p* < 0.05).

Consistent with the DA results, P1 received higher intensity ratings for “brown color”, whereas P6 was rated the highest for “yellow color”. Moreover, “sweet pumpkin_F” and “sweet pumpkin_A” were also rated the highest for P2, and “sweet_F” was rated highly for P1, P2, and P6. The results for the textural attribute “thickness” were in contrast to that obtained with DA, as P4 received the lowest ratings for “thickness” in RATA (Table 6).

**Figure 3:**
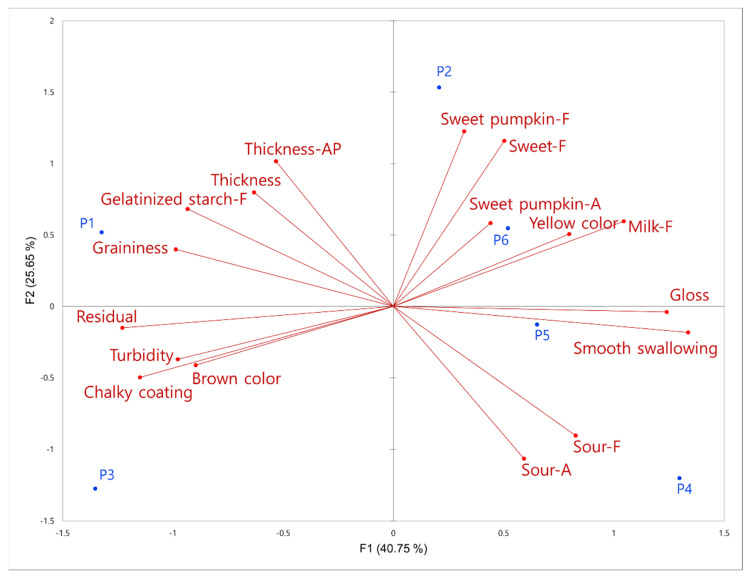
Principal component analysis of the significantly different rate-all-that-apply (RATA) characteristics. AP, F, and A at the end of attribute indicate appearance, flavor, and aroma, respectively.

We performed a PCA using significant characteristics obtained from the RATA (Figure 3). It presented 69.54% of the total variance in a second dimension map, where the samples were divided into four groups associated with the following attributes: P1 sample with “gelatinized starch_F”, “thickness_AP”, and “graininess”; P2, P5, and P6 samples with “sweet_F”, “sweet pumpkin_F”, “gloss”, “smooth swallowing”, “milk_F”, “sweet pumpkin_A”, and “yellow color”; P3 sample with “brown color” and “turbidity”; and P4 sample with “sour_F” and “sour_A”. These results are consistent with the grouping tendencies observed in the DA results and are similar to the CA plot of CATA.

### 3.4. Flash Profile

During the first session of FP, each participant individually generated 5–15 sensory attributes for six samples of sweet pumpkin porridge, resulting in a total of 458 sensory terms obtained from 60 participants. Among all of the terms, 51 terms showed significant semantic differences (*p* < 0.05), including all 20 terms generated by the DA panelists (Table 7). This indicates that untrained consumers could generate terms with similar abilities to the DA panelists when describing sweet pumpkin porridge [58]. This could be possible because the consumers were instructed to avoid hedonic or unrelated sensory terms for the samples as is usually performed by trained panelists.

**Figure 4:**
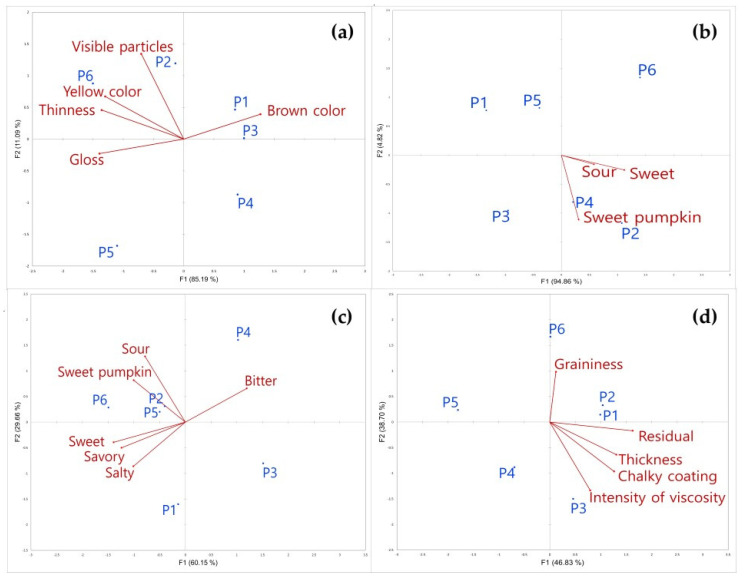
Principal component analysis (PCA) plots of the sensory attributes from the generalized procrustes analysis (GPA): (**a**) appearance; (**b**) aroma; (**c**) flavor; (**d**) mouthfeel.

PCA plots were created using the GPA data obtained from the FP evaluation (Figure 4). To enhance the data visualization, the map was divided into appearance, aroma, flavor, and texture attributes. The appearance attributes consisted of “brown color”, “thinness”, “yellow color”, “visible particles”, and “gloss”, with P1 and P3 grouped based on “brown color”. P2 exhibited a significant correlation with “visible particles”, whereas P6 was characterized by “thinness” and “yellow color” (Figure 4a). This outcome generally aligned with the DA results, further indicating that consumers could distinguish the samples based on sensory perception [58]. This might illustrate the advantage of FP in identifying the most significant and distinguishing attributes of the product. This feature might be valuable in the initial phases of other consumer evaluations that demand established profiles or attribute questionnaires [58].

For the aroma attributes, “sour”, “sweet”, and “sweet pumpkin” were identified as significant attributes (*p* < 0.05). P2 and P4 demonstrated a strong correlation with “sweet pumpkin”. Except for P4, the remaining samples did not display significant correlations with any other aroma attributes (Figure 4b). Additionally, the number of significant aroma attributes in the PCA was notably lower when compared with other sensory modalities (Figure 4).

The reasons for this observation could be twofold. First, identifying aroma-related attributes of the samples might not have been an easy task for consumers. It has been reported that understanding the definition and detecting aroma sensory attributes requires more time and effort compared with other sensory descriptions, such as related attributes [57]. Second, as previously mentioned, this could be attributed to the loss of volatile aroma compounds during the processing of pumpkin porridge, leading to consumers having difficulty in perceiving the aroma of the provided samples [49]. It is well known that the intense heat treatment of food can lead to a reduction in volatile components within food products.

For the flavor attributes (Figure 4c), “sweet pumpkin”, “savory”, “salty”, “bitter”, “sweet”, and “sour” were significant (*p* < 0.05). P4 exhibited a strong correlation with the “bitter” flavor, whereas P2 was associated with “sweet pumpkin”, and P1 was related to the “savory”, “salty”, and “sweet” flavors. Significant mouthfeel attributes included “graininess”, “residual”, “thickness”, “chalky coating”, and “intensity of viscosity”, where “graininess” was strongly correlated with P6, and all mouthfeel attributes, except “graininess”, were associated with P1 and P3 (Figure 4d). As observed in the RATA results, the attributes “gelatinized starch_A” and “gelatinized starch_F” were not significantly mentioned by consumers in the FP. This further demonstrates that these attributes were difficult for consumers to define.

Interestingly, contradictory observations between these two methods were demonstrated. For example, “visible particles” was not a main attribute in the DA, yet consumers mentioned it relatively frequently among the appearance attributes (Table 7). This could potentially indicate that the DA panelists may have missed attributes that were perceptible to consumers [12]. The texture attributes derived from the DA amounted to a total of five, whereas consumers using FP generated a comparatively greater number of texture attributes. For instance, “smooth swallowing” emerged as a significant attribute in both the FP and DA. However, consumers additionally identified “softness”, implying that they took into account not only the sensation while swallowing but also the immediate feeling upon introducing the product into their mouth.

### 3.5. Free Listing

In the FL, participants provided a minimum of 10 and a maximum of 72 attributes for each sweet pumpkin porridge sample, resulting in a total of 1473 terms obtained from 60 consumers. The prescreening process for the term selection was conducted by analyzing only the attributes mentioned by more than 10% of the participants. A total of 35 descriptors representing the appearance, aroma, flavor, and texture were developed.

In FL tasks, term significance derives from the following two factors: its mention frequency and position in the consumer-provided sensory descriptor list. Combining these factors yields a meaningful assessment of the importance of each term in the opinion of consumers. The attributes with the greatest significance are mentioned more by consumers and are ranked higher [46]. However, relying solely on these factors yields differing insights [47]. Thus, a quantitative parameter is vital to capture both effectively, necessitating the utilization of the SSI [46].

P1 and P6 showed relatively high values among all samples, indicating their closest association with “brown color” and “yellow color”, respectively (Table 8). Additionally, color attributes exhibited relatively high SSI values compared with other attributes. This suggests that consumers can perceive color relatively easily compared with other attributes. All samples exhibited high SSI values in similar patterns, along with corresponding citation frequencies, for the attributes “sweet pumpkin_A” and “sweet_F”. This implies that consumers were able to detect these two main attributes effectively across all samples. Regarding the texture attributes, P2 had a high SSI value for “thickness”, whereas P4 showed a high value for “thinness”, displaying a different pattern from the DA results. However, this trend was similar to the results of the consumer-based methods, namely, CATA, RATA, and FP. This again highlights the difference in discriminative ability for the “viscosity” between the trained panels and untrained consumers.

**Figure 5:**
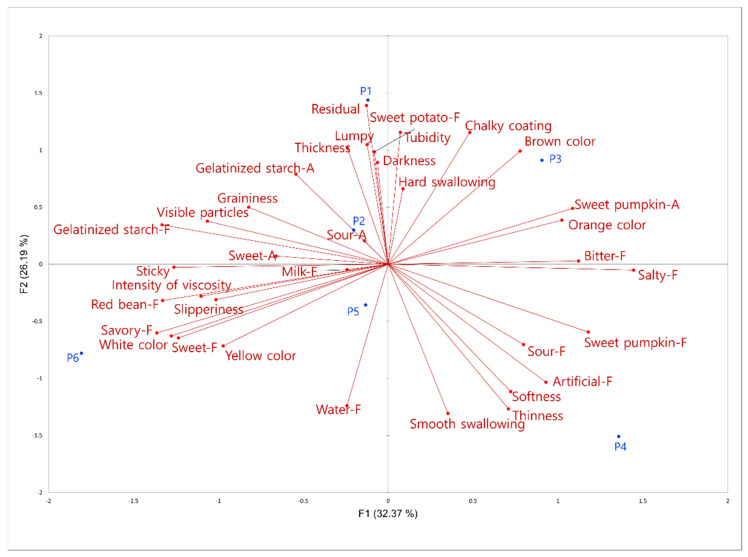
Principal component analysis (PCA) plot displaying the first two principal components performed on the Smith salience index (SSI) values from the free listing. AP, F, and A at the end of an attribute indicate appearance, flavor, and aroma, respectively.

Figure 5 represents the PCA plot using the SSI values, wherein the sensory attribute terms derived from the FL are more elaborate than those originating from the DA, resulting in a more accurate reflection of consumer perceptions. Unlike DA and other consumer-based methods, FL did not show clear groupings for P2, P5, and P6. This suggests that consumers were able to extract more distinctive attributes for each sample in their own language, making the grouping less evident.

### 3.6. Comparison of the DA and Consumer-Based Sensory Profiling

According to Fleming et al. [59], the congruence of the configurations and the discriminative power of different methodologies can be observed through dendrograms generated using hierarchical cluster analysis (HCA), visual plots generated using MFA, and Rv coefficients.

**Figure 6:**
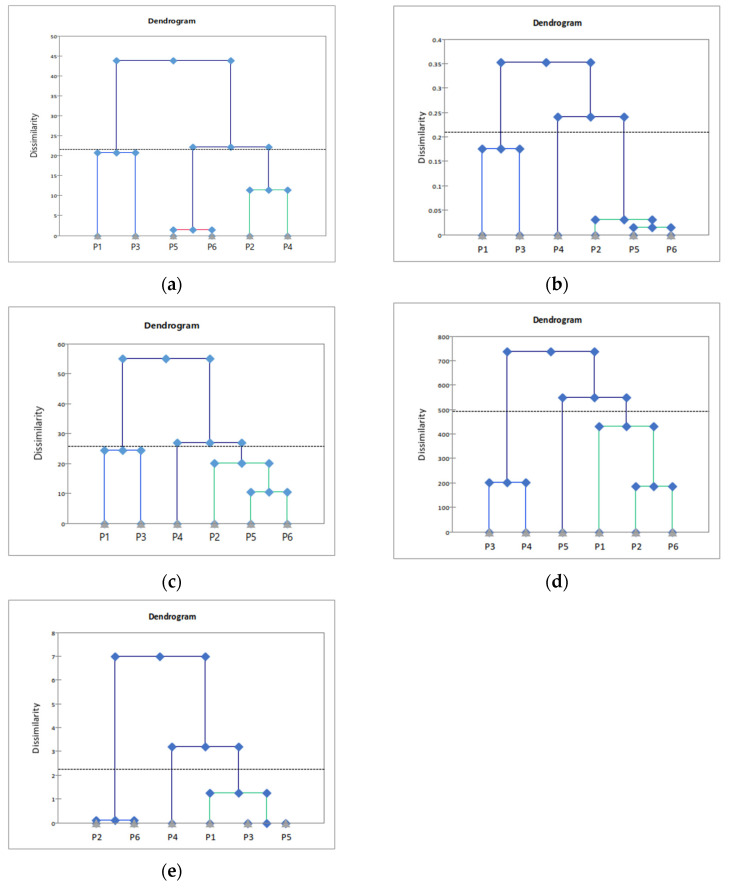
Dendrogram of the hierarchical cluster analysis (HCA) of the samples assessed using (**a**) descriptive analysis; (**b**) check-all-that-apply; (**c**) rate-all-that-apply; (**d**) flash profile: (**e**) free listing.

Figure 6 shows the HCA dendrogram of the samples obtained through DA and four consumer-based sensory profiling methods. This allows for the identification of groups with similar or different sensory attributes. DA, CATA, and RATA grouped the samples into three clusters. The three methods grouped P1, P3, and P5 together, as well as P6, based on the visual attribute of color. In contrast, FP and FL showed slightly different grouping patterns compared with DA. The FP grouped the samples into two clusters, viz., P3 and P4, and P1, P2, and P6, driven by color and texture attributes. This indicates that both trained panelists and untrained consumers could distinguish samples effectively based on color.

Methods with Rv values above 0.9 indicate a relatively high similarity among each other in relation to product configurations and sensory characteristics. FL showed the lowest Rv value of 0.89 with DA, whereas the other three methods had Rv values of 0.9 or above, with RATA, CATA, and FP in descending order (Rv = 0.96, 0.91, and 0.91, respectively). Among the different methods, RATA exhibited the highest Rv value of 0.958, indicating the highest similarity to DA.

The superiority of RATA as an alternative to DA in assessing samples with complex or subtle differences has been demonstrated in some studies [29]. This enhanced feature comes from the quantification of the intensity of sensory attributes. In this study, RATA demonstrated significant differences (*p* < 0.05) for the “sweet pumpkin_A” and “sweet_F” attributes and effectively identified samples that were closely characterized with these attributes. On the contrary, although both “sweet pumpkin_A” and “sweet_F” exhibited high SSI values across all samples in FL, it was not possible to determine which samples were quantitatively associated with these attributes. This limitation arises from the fact that the SSI does not take into account the significance of differences in attributes, and the data analysis with the FL is more intricate compared with that using other sensory methods [23]. Yoon et al. [27] reported the lowest Rv value for FL using DA for RTD coffee beverages compared with CATA and FP. In the analysis of the FL data, the terminology used by individual consumers needs to be refined and standardized before conducting a statistical analysis.

### 3.7. Feedback Questionnaire

The evaluation times were calculated by subtracting the evaluation start time from the evaluation end time. The levels of tediousness and difficulty were requested as the final items on the questionnaire. The results for these three evaluations are presented in Table 9. As revealed by using the ANOVA, the FP evaluation required significantly more time compared with the CATA and RATA methods (*p* < 0.05). This could be due to the evaluation being conducted across two sessions, and it also yielded significantly higher values for tediousness and difficulty compared with other methods. In contrast, both the CATA and RATA methods had much shorter evaluation times, lower difficulty levels, and significantly lower levels of tediousness. These findings align with previous reports wherein the advantages of the CATA and RATA methods were discussed in terms of the ease of task and time efficiency. Additionally, there is a perception that RATA is more tedious than CATA [60]. However, we did not find any significant difference in the level of tediousness between CATA and RATA (*p* < 0.05).

## 4. Conclusions

The four consumer-based sensory profiling methods (CATA, RATA, FP, and FL) evaluated in this study could be good alternatives to conventional DA for the sensory profiling of sweet pumpkin porridge. Among these methods, RATA showed the highest similarity to DA, with an Rv value of 0.958. Thus, RATA might be a more effective approach than any other consumer-based methods when assessing similar products based on their sensory attributes, owing to its ability to quantify attribute intensity. Given its features of simplicity, ease, and versatility in evaluating consumer perception, CATA might be a more effective tool in profiling the sensory attributes of products with medium to large sensory differences, which can be easily categorized into groups. While the FP and FL methods may demand more time and resources compared with CATA and RATA, because of the need to refine and generalize the terminology used by individual consumers, these methods warrant consideration. This is because they allow consumers to directly articulate product-related sensory attributes, and they possess the advantage of pinpointing the subjective perspectives of consumers on the product. Future studies should consider exploring the application of hybrid methods that combine the advantages of CATA and RATA to reflect the spontaneous perception of products by consumers based on FP or FL.

## Figures and Tables

**Table 1:** Product description of the sweet pumpkin porridge samples.

Samples	Sweet Pumpkin Content (%) and Origin	Ingredients
P1	24 (China)	Sweet pumpkin dice 8%, chestnut dice 4.2%, soybean 4%, sugar, etc.
P2	26.42 (China)	Pumpkin 10.71%, red bean flakes 1.87%, rice flour, sugar, etc.
P3	43 (China)	Powdered cream (cream, refined salt), specified honey 0.5%, sugar, etc.
P4	48.89 (China)	Sweet pumpkin paste [sweet pumpkin puree, white bean paste (soybean paste)], red bean 1.48%, sugar processed products, xanthan gum, sugar, etc.
P5	40 (China)	Sweet pumpkin paste, mixed milk, sugar, etc.
P6	34 (Foreign origin/Vietnam, China, New Zealand, etc.)	Sweet red bean paste 3.8% (red bean 51%, sugar), rice grits, vitamin C, sugar, etc.

Product descriptions are as they are on the package.

**Table 2:** Attributes and their references for sweet pumpkin porridge samples.

	Attribute	Definition	Reference (Intensity)
Appearance	Yellow color	Intensity of the yellow color of sweet pumpkin porridge	-
	Brown color	Intensity of the brown color of sweet pumpkin porridge	-
	Appearance of viscosity	Intensity of the appearance of the viscosity of sweet pumpkin porridge	-
	Gloss	Intensity of the gloss of sweet pumpkin porridge	-
	Turbidity	Intensity of the turbidity of sweet pumpkin porridge	-
Aroma	Sweet pumpkin	Intensity of the sweet pumpkin aroma of sweet pumpkin porridge	Steamed sweet pumpkin (9)
	Sweet	Intensity of the sweet aroma of sweet pumpkin porridge	Granola cereal (6) (Kellogg, Battle Creek, MI, USA)
	Gelatinized starch	Intensity of the gelatinized starch aroma of sweet pumpkin porridge	Rice gruel (Hetbahn, CJ Cheiljedang, Seoul, Republic of Korea) (6)
	Sour	Intensity of the sour aroma of sweet pumpkin porridge	Minced dill pickles (4.5) (Rio santo, YONCA FOOD, Turkiye)
Flavor	Sweet	Intensity of the sweet taste of sweet pumpkin porridge	2%, 4% Sucrose solution (4), (6)
	Salty	Intensity of the salty taste of sweet pumpkin porridge	2%, 4% NaClsolution (3), (5)
	Sour	Intensity of the sour taste of sweet pumpkin porridge	2.5%, 4% citric acid solution (3), (5)
	Gelatinized starch	Intensity of the gelatinized starch flavor of sweet pumpkin porridge	Rice gruel (Hetbahn, CJ Cheiljedang, Seoul, Republic of Korea) (8)
	Sweet pumpkin	Intensity of the sweet pumpkin flavor of sweet pumpkin porridge	Steamed sweet pumpkin (9)
	Milk	Intensity of the milk flavor of sweet pumpkin porridge	Whole milk (7.5) (Seoul Milk, Seoul, Republic of Korea)
Mouthfeel	Chalky coating	Intensity of the chalky coating of milk of sweet pumpkin porridge	Greek yogurt, plain (5.5) (Ildong Foodis Co., Ltd., Republic of Korea)
	Residual	Intensity of the aroma or flavor remaining in the mouth after tasting sweet pumpkin porridge	Carrot cube, 2 × 2 cm (5)
	Smooth swallowing	Intensity of the smooth swallowing of sweet pumpkin porridge	Water (8), Yogurt (13) (Maeil Dairy Industry Co., Ltd., Republic of Korea)
	Viscosity	Intensity of the thickness felt when holding the sweet pumpkin porridge in the mouth	Whipped cream (5.5) (Président, France)
	Graininess	Grains chewed using teeth when tasting sweet pumpkin porridge	Sweet pumpkin porridge (3.5) (Somemeal, Ganghwadream Co., Ltd., Republic of Korea), Sweet pumpkin porridge (Choroc Co., Ltd., Republic of Korea) (6.5)

**Table 3:** Mean values of significant descriptive sensory attributes for the sweet pumpkin porridge samples.

Attribute	P1	P2	P3	P4	P5	P6	*p*-Value
Appearance							
Yellow color	6.2 ^d^	8.4 ^b^	6.0 ^d^	7.3 ^c^	11.0 ^a^	11.4 ^a^	*<0.0001*
Brown color	8.4 ^a^	4.3 ^d^	7.0 ^b^	5.9 ^c^	2.5 ^e^	0.4 ^f^	*<0.0001*
Appearance Viscosityt	8.0 ^ab^	7.6 ^b^	8.4 ^a^	8.1 ^ab^	4.8 ^c^	4.9 ^c^	*<0.0001*
Gloss	5.0 ^c^	5.1 ^c^	3.7 ^d^	8.0 ^a^	7.1 ^b^	7.6 ^ab^	*<0.0001*
Turbidity	4.9 ^b^	4.8 ^b^	6.5 ^a^	4.0 ^c^	4.5 ^bc^	2.5 ^d^	*<0.0001*
Aroma							
Sweet pumpkin	7.8 ^b^	10.0 ^a^	6.8 ^c^	8.4 ^b^	8.2 ^b^	6.0 ^d^	*<0.0001*
Sweet	5.5 ^a^	4.7 ^b^	4.0 ^c^	4.3 ^bc^	4.9 ^ab^	4.8 ^b^	*<0.0001*
Gelatinized starch	2.5 ^a^	1.1 ^b^	2.8 ^a^	2.2 ^a^	1.5 ^b^	2.2 ^a^	*<0.0001*
Sour	0.5 ^c^	1.5 ^b^	2.7 ^a^	3.0 ^a^	1.5 ^b^	1.8 ^b^	*<0.0001*
Flavor							
Sweet	7.6 ^a^	7.8 ^a^	2.3 ^d^	5.6 ^c^	6.5 ^b^	7.8 ^a^	*<0.0001*
Salty	1.1 ^cd^	1.5 ^c^	3.0 ^a^	2.1 ^b^	2.4 ^b^	1.0 ^d^	*<0.0001*
Sour	0.4 ^d^	2.1 ^c^	3.3 ^b^	5.1 ^a^	2.3 ^c^	1.9 ^c^	*<0.0001*
Gelatinized starch	2.5 ^b^	3.1 ^ab^	3.2 ^a^	0.8 ^c^	1.2 ^c^	2.8 ^ab^	*<0.0001*
Sweet pumpkin	7.4 ^b^	9.9 ^a^	4.9 ^d^	6.5 ^c^	7.0 ^bc^	6.4 ^c^	*<0.0001*
Milk	0.6 ^c^	1.8 ^a^	0.4 ^c^	1.3 ^b^	2.1 ^a^	1.1 ^b^	*<0.0001*
Mouthfeel							
Chalky coating	6.4 ^b^	2.1 ^d^	8.1 ^a^	1.3 ^e^	5.6 ^c^	2.3 ^d^	*<0.0001*
Residual	8.2 ^a^	1.9 ^e^	6.6 ^b^	1.7 ^e^	4.5 ^c^	3.6 ^d^	*<0.0001*
Smooth swallowing	2.5 ^c^	7.6 ^b^	3.1 ^c^	11.9 ^a^	7.0 ^b^	6.8 ^b^	*<0.0001*
Viscosity	6.9 ^bc^	7.9 ^a^	7.6 ^ab^	7.9 ^a^	5.9 ^d^	6.3 ^cd^	*<0.0001*
Graininess	9.5 ^a^	2.0 ^d^	3.4 ^c^	1.1 ^e^	2.6 ^d^	4.9 ^b^	*<0.0001*

The same letter within the same row are not significantly different (*p* < 0.05).

**Table 4:** Frequency of the sensory attributes using the check-all-that-apply (CATA) method for the sweet pumpkin porridge samples.

Attribute	P1	P2	P3	P4	P5	P6	*p*-Value
Appearance							
Brown color	49 ^a^	28 ^b^	36 ^ab^	30 ^b^	5 ^c^	0 ^c^	*<0.0001*
Yellow color	30 ^b^	49 ^a^	28 ^b^	29 ^b^	59 ^a^	60 ^a^	*<0.0001*
Gloss	32 ^cd^	33 ^bcd^	25 ^d^	48 ^a^	43 ^abc^	46 ^ab^	*<0.0001*
Turbidity	30 ^a^	29 ^a^	36 ^a^	23 ^ab^	26 ^ab^	13 ^b^	*<0.0001*
Thickness	47 ^a^	41 ^ab^	51 ^a^	48 ^a^	24 ^c^	30 ^bc^	*<0.0001*
Aroma							
Sweet pumpkin	45 ^ab^	56 ^a^	38 ^b^	48 ^ab^	46 ^ab^	35 ^b^	*<0.0001*
Sweet	33 ^a^	26 ^a^	19 ^a^	21 ^a^	28 ^a^	27 ^a^	*0.081*
Sour	5 ^b^	16 ^ab^	23 ^a^	25 ^a^	16 ^ab^	19 ^ab^	*0.001*
Gelatinized starch	25 ^ab^	12 ^b^	28 ^a^	23 ^ab^	18 ^ab^	23 ^ab^	*0.029*
Flavor							
Sweet pumpkin	40 ^abc^	54 ^a^	28 ^c^	39 ^bc^	44 ^ab^	37 ^bc^	*<0.0001*
Sweet	43 ^a^	45 ^a^	17 ^c^	28 ^bc^	36 ^ab^	45 ^a^	*<0.0001*
Salt	7 ^ab^	10 ^ab^	18 ^a^	13 ^ab^	16 ^ab^	6 ^b^	*0.012*
Sour	1 ^c^	16 ^ab^	22 ^ab^	26 ^a^	13 ^abc^	12 ^bc^	*<0.0001*
Gelatinized starch	15 ^ab^	21 ^a^	23 ^a^	7 ^b^	11 ^ab^	19 ^ab^	*0.003*
Milk	5 ^a^	10 ^a^	4 ^a^	9 ^a^	14 ^a^	8 ^a^	*0.090*
Mouthfeel							
Residual	48 ^a^	14 ^c^	37 ^ab^	13 ^c^	27 ^bc^	19 ^c^	*<0.0001*
Chalky coating	36 ^a^	13 ^b^	42 ^a^	6 ^b^	32 ^a^	15 ^b^	*<0.0001*
Thickness	36 ^ab^	42 ^a^	27 ^b^	9 ^c^	24 ^b^	37 ^ab^	*<0.0001*
Graininess	49 ^a^	10 ^bc^	17 ^bc^	4 ^c^	13 ^bc^	23 ^b^	*<0.0001*
Smooth swallowing	12 ^d^	38 ^b^	18 ^cd^	55 ^a^	35 ^b^	32 ^bc^	*<0.0001*

The same letter within the same row are not significantly different (*p* < 0.05).

**Table 5:** Relationships between the attributes and samples based on multiple discriminant analysis (MDA) using the check-all-that-apply (CATA) method.

Attribute	P1	P2	P3	P4	P5	P6
Appearance						
Brown color	+0.511	−0.075	+0.495	+0.058	−0.725 ^b^	−0.830 ^b^
Yellow color	−0.452	+0.280	−0.613	−0.127	+0.703	+0.828 ^a^
Gloss	−0.628	−0.040	−0.636	+0.622	+0.331	+0.657
Turbidity	+0.137	−0.021	+0.786 ^a^	−0.061	−0.191	−0.906 ^b^
Thickness	−0.042	−0.084	+0.578	+0.523	−0.876 ^b^	−0.533
Aroma						
Sweet pumpkin	−0.490	+0.717 ^a^	−0.440	+0.566	+0.098	−0.137
Sweet	+0.361	+0.010	−0.830 ^b^	−0.412	+0.485	+0.551
Sour	−0.908 ^b^	+0.000	+0.221	+0.775 ^a^	−0.083	+0.209
Gelatinized starch	−0.008	−0.835 ^b^	+0.498	+0.228	−0.312	+0.038
Flavor						
Sweet pumpkin	−0.397	+0.804 ^a^	−0.768 ^b^	+0.228	+0.345	+0.304
Sweet	+0.038	+0.459	−0.908 ^b^	−0.200	+0.264	+0.719 ^a^
Salt	−0.508	−0.212	+0.580	+0.348	+0.270	−0.429
Sour	−0.864 ^b^	+0.109	+0.298	+0.841 ^a^	−0.173	−0.062
Gelatinized starch	−0.030	+0.279	+0.420	−0.443	−0.264	+0.202
Milk	−0.585	+0.319	−0.631	+0.283	+0.782 ^a^	+0.373
Mouthfeel						
Residual	+0.813 ^a^	−0.690	+0.502	−0.658	−0.039	−0.426
Chalky coating	+0.509	−0.579	+0.658	−0.663	+0.216	−0.416
Thickness	+0.280	+0.465	−0.213	−0.702	+0.003	+0.431
Graininess	+0.915 ^a^	−0.436	−0.024	−0.705	−0.155	+0.008
Smooth swallowing	−0.833 ^b^	+0.326	−0.441	+0.875 ^a^	+0.117	+0.295

^a^ Means high positive correlation between each sample and the sensory attributes. ^b^ Means high negative correlation between each sample and the sensory attributes.

**Table 6:** Mean values of significant rate-all-that-apply (RATA) sensory attributes for the sweet pumpkin porridge samples.

Attribute	P1	P2	P3	P4	P5	P6	*p*-Value
Appearance							
Brown color	2.9 ^a^	1.5 ^cd^	2.4 ^ab^	2.0 ^bc^	0.8 ^de^	0.1 ^e^	*<0.0001*
Yellow color	2.2 ^bc^	3.0 ^b^	2.2 ^c^	2.5 ^bc^	3.9 ^a^	4.0 ^a^	*<0.0001*
Gloss	1.8 ^bc^	1.8 ^abc^	1.4 ^c^	2.7 ^a^	2.4 ^ab^	2.6 ^ab^	*<0.0001*
Turbidity	1.8 ^ab^	1.8 ^ab^	2.2 ^a^	1.4 ^ab^	1.6 ^ab^	1.0 ^b^	*0.014*
Thickness	1.9 ^bcd^	3.2 ^a^	2.6 ^ab^	1.1 ^d^	1.5 ^cd^	2.1 ^bc^	*<0.0001*
Aroma							
Sweet pumpkin	2.7 ^ab^	3.4 ^a^	2.3 ^b^	2.9 ^ab^	2.8 ^ab^	2.2 ^b^	*0.000*
Sweet	1.9 ^a^	1.6 ^a^	1.5 ^a^	1.6 ^a^	1.7 ^a^	1.7 ^a^	*0.805*
Sour	0.2 ^b^	0.5 ^ab^	0.9 ^a^	1.0 ^a^	0.6 ^ab^	0.7 ^ab^	*0.006*
Gelatinized starch	0.8 ^a^	0.5 ^a^	1.0 _a_	0.8 ^a^	0.6 ^a^	0.7 _a_	*0.412*
Flavor							
Sweet pumpkin	2.5 ^b^	3.4 ^a^	1.8 ^b^	2.2 ^b^	2.4 ^b^	2.3 ^b^	*<0.0001*
Sweet	2.6 ^a^	2.7 ^a^	0.9 ^b^	2.0 ^a^	2.3 ^a^	2.7 ^a^	*<0.0001*
Salt	0.4 ^a^	0.6 ^a^	1.0 ^a^	0.7 ^a^	0.8 ^a^	0.4 ^a^	*0.101*
Sour	0.1 ^c^	0.8 ^b^	1.1 ^ab^	1.7 ^a^	0.9 ^b^	0.7 ^bc^	*<0.0001*
Gelatinized starch	0.8 ^a^	1.0 ^a^	1.0 ^a^	0.3 ^a^	0.5 ^a^	0.9 ^a^	*0.019*
Milk	0.2 ^ab^	0.6 ^a^	0.1 ^b^	0.4 ^ab^	0.7 ^a^	0.3 ^ab^	*0.007*
Mouthfeel							
Residual	2.9 ^a^	0.8 ^d^	2.3 ^ab^	0.7 ^d^	1.6 ^bc^	1.4 ^cd^	*<0.0001*
Chalky coating	2.2 ^ab^	0.7 ^c^	2.8 ^a^	0.5 ^c^	1.9 ^b^	0.8 ^c^	*<0.0001*
Thickness	1.7 ^bc^	2.8 ^a^	1.8 ^b^	0.7 ^d^	0.8 ^cd^	2.0 ^ab^	*<0.0001*
Graininess	3.3 ^a^	0.7 ^cd^	1.3 ^bc^	0.4 ^d^	0.8 ^cd^	1.7 ^b^	*<0.0001*
Smooth swallowing	0.9 ^c^	2.6 ^b^	1.1 ^c^	4.1 ^a^	2.5 ^b^	2.4 ^b^	*<0.0001*

The same letter within the same row are not significantly different (*p* < 0.05).

**Table 7:** Attributes and their frequencies mentioned in the flash profile of sweet pumpkin porridge samples.

Attribute	Number of Citations	Attribute	Number of Citations
Brightness	3	Gelatinized starch_F *	3
Brown color *	23	Light_F	1
Gloss *	12	Milk_F *	1
Intensity of viscosity *	1	Nutty_F	1
Orange color	1	Salty_F	5
Thickness *	6	Savory_F	6
Thinness *	18	Sour_F	29
Transparent	3	Sweet potato_F	1
Turbidity *	2	Sweet pumpkin_F *	21
Visible particles	14	Sweet_F *	54
White color	1	After aroma	1
Yellow color *	30	After taste	1
Burnt_A	1	Astringent	6
Curry_A	1	Chalky coating *	24
Gelatinized starch_A *	1	Graininess *	37
Mustard_A	1	Intensity of viscosity *	14
Savory_A	1	Lumpy	5
Sour_A *	2	Moistness	3
Sweet pumpkin_A *	24	Residual *	28
Sweet_A *	10	Slipperiness	1
Bean_F	1	Smooth swallowing *	4
Bitter_F	14	Softness	6
Burnt_F	1	Sticky *	4
Butter_F	1	Thickness *	26
Fatty_F	2		
Fishy_F	1		
Fresh_F	1		

* Sensory attribute terms used by the descriptive analysis panel. F, and A at the end of attribute indicate flavor, and aroma, respectively.

**Table 8:** SSI, number of citations, and average positions for the attributes of sweet pumpkin porridge samples for the free listing.

Attribute	P1			P2			P3			P4			P5			P6		
	SSI ^1^	N ^2^	AP ^3^	SSI	N	AP	SSI	N	AP	SSI	N	AP	SSI	N	AP	SSI	N	AP
Appearance																		
Brown color	0.265	22	2.5	0.080	8	2.9	0.169	17	3.0	0.133	12	2.5	0.022	4	3.8	0.007	1	4.0
Orange color	0.015	1	2.0	0.028	2	1.5	0.023	2	3.5	0.023	3	4.3	-	-	-	-	-	-
Turbidity	0.041	4	2.5	0.004	1	4.0	0.037	3	2.7	0.006	1	3.0	0.002	2	7.0	0.017	1	1.0
Visible particles	0.148	12	2.8	0.160	14	2.2	0.012	1	3.0	0.025	2	2.0	0.007	1	4.0	0.161	14	2.7
Yellow color	0.272	21	2.1	0.187	16	2.4	0.280	20	1.9	0.301	23	2.0	0.434	35	2.0	0.544	40	1.9
White color	-	-	-	-	-	-	-	-	-	-	-	-	0.010	1	3.0	0.027	3	2.5
Darkness	0.017	1	1.0	-	-	-	0.029	3	4.0	-	-	-	-	-	-	0.015	1	2.0
Aroma																		
Gelatinized starch	0.036	4	3.8	0.070	7	3.3	0.061	4	2.0	0.011	1	2.0	0.025	2	2.5	0.051	5	2.8
Sour	-	-	-	0.011	3	2.0	-	-	-	-	-	-	-	-	-	-	-	-
Sweet pumpkin	0.233	18	2.3	0.285	20	2.0	0.298	22	2.2	0.252	20	2.2	0.272	19	1.8	0.177	16	3.1
Sweet	0.066	6	3.0	0.122	9	2.0	0.041	3	2.7	0.054	4	1.8	0.049	4	2.5	0.078	5	1.8
Flavor																		
Artificial	-	-	-	-	-	-	-	-	-	0.003	3	5.0	-	-	-	-	-	-
Bitter	0.045	4	2.5	0.033	3	3.7	0.055	5	2.4	0.071	7	2.9	-	-	-	0.017	1	1.0
Gelatinized starch	0.044	5	3.6	0.035	4	3.8	0.008	1	5.0	0.007	1	5.0	0.017	2	4.0	0.052	8	5.4
Milk	0.028	2	2.0	0.011	1	2.0	0.024	2	3.0	0.010	2	3.5	0.102	9	2.7	0.025	2	2.0
Red bean	0.011	1	2.0	-	-	-	-	-	-	-	-	-	0.017	1	1.0	031	3	4.3
Salty	0.033	4	5.0	0.038	6	4.5	0.081	7	3.3	0.078	7	2.6	0.033	4	4.0	0.010	2	7.0
Savory	-	-	-	0.011	1	2.0	-	-	-	-	-	-	0.017	2	1.0	0.037	3	2.3
Sour	-	-	-	0.099	11	3.5	0.144	15	3.6	0.132	12	2.8	0.111	12	3.6	0.069	8	4.0
Sweet potato	0.013	3	6.5	-	-	-	-	-	-	-	-	-	-	-	-	-	-	-
Sweet pumpkin	0.180	19	3.0	0.152	18	3.2	0.263	21	2.7	0.292	29	2.6	0.232	23	2.8	0.177	19	3.3
Sweet	0.319	32	3.0	0.346	33	2.7	0.229	21	3.2	0.309	30	2.7	0.340	29	2.3	0.399	41	2.9
Water	-	-	-	-	-	-	-	-	-	0.030	2	2.7	-	-	-	0.030	3	1.5
Mouthfeel																		
Chalky coating	0.135	15	3.3	0.074	6	2.0	0.199	19	3.5	0.033	4	3.3	0.137	16	3.6	0.034	3	2.0
Graininess	0.166	20	3.3	0.017	1	1.0	0.049	5	3.2	0.030	3	3.0	0.114	12	3.3	0.123	10	3.2
Hard swallowing	0.015	2	5.5	0.003	1	6.0	0.009	1	2.0	-	-	-	0.023	5	4.8	-	-	-
Intensity of viscosity	-	-	-	0.013	1	2.0	0.015	2	3.5	-	-	-	0.017	1	1.0	0.030	3	2.7
Lumpy	0.074	5	1.4	0.017	2	2.5	-	-	-	-	-	-	0.006	1	5.0	-	-	-
Residual	0.041	5	4.8	0.032	3	2.3	0.032	5	5.0	0.018	3	3.7	0.033	5	4.0	0.023	4	4.0
Slipperiness	-	-	-	0.027	2	2.5	-	-	-	0.002	1	8.0	-	-	-	0.025	3	4.7
Smooth swallowing	0.017	1	1.0	0.036	5	2.8	0.009	1	2.0	0.063	8	3.5	0.044	4	2.3	0.030	4	4.8
Softness	0.017	2	3.5	0.031	4	3.3	0.006	1	3.0	0.070	7	3.1	0.026	4	4.0	0.017	2	3.5

^1^ Smith salience index. ^2^ Number of citations attributes. ^3^ Average position.

**Table 9:** Comparison of the efficiency, difficulty, and tediousness of consumer-based sensory profiling methods.

	Time to Complete (min) ^1^	Difficulty of Test	Tediousness of Test
CATA	11.7 ^c^	2.5 ^b^	1.6 ^bc^
RATA	15.8 ^b^	2.5 ^b^	1.4 ^c^
FP	15.8 ^b^	2.6 ^b^	1.7 ^ab^
FL	22.7 ^a^	3.1 ^a^	2.0 ^a^
Pr > F (Model)	*<0.0001*	*0.019*	*0.000*

^1^ Average time to complete each test was evaluated during consumer test. The same letter within the same column are not significantly different (*p* < 0.05). The difficulty and tediousness of the test was assessed using 5-point scales (1: low; 3: medium, and 5: high).

## Data Availability

The data that support the findings of this study are available from the corresponding author upon reasonable request.
